# Combined p21-activated kinase and farnesyltransferase inhibitor treatment exhibits enhanced anti-proliferative activity on melanoma, colon and lung cancer cell lines

**DOI:** 10.1186/1476-4598-12-88

**Published:** 2013-08-06

**Authors:** Giampiero Porcu, Ainslie B Parsons, Daniele Di Giandomenico, Giuseppe Lucisano, Maria Giovanna Mosca, Charles Boone, Antonella Ragnini-Wilson

**Affiliations:** 1 Department of Translational Pharmacology, Consorzio Mario Negri Sud, S. Maria Imbaro, Italy; 2 Department of Molecular Genetics, Donnelly Centre, University of Toronto, 160 College Street, Toronto, Ontario M5S 3E1, Canada; 3 Department of Clinical Pharmacology and Epidemiology, Consorzio Mario Negri Sud, S. Maria Imbaro, Italy; 4 Department of Biology, University of Rome Tor Vergata, Rome, Italy

**Keywords:** Farnesylation, PAKs, Cancer, Yeast, Anti-cancer drug screening

## Abstract

**Background:**

Farnesyltransferase inhibitors (FTIs) are anticancer agents with a spectrum of activity in Ras-dependent and independent tumor cellular and xenograph models. How inhibition of protein farnesylation by FTIs results in reduced cancer cell proliferation is poorly understood due to the multiplicity of potential FTase targets. The low toxicity and oral availability of FTIs led to their introduction into clinical trials for the treatment of breast cancer, hematopoietic malignancy, advanced solid tumor and pancreatic cancer treatment, and Hutchinson-Gilford Progeria Syndrome. Although their efficacy in combinatorial therapies with conventional anticancer treatment for myeloid malignancy and solid tumors is promising, the overall results of clinical tests are far below expectations. Further exploitation of FTIs in the clinic will strongly rely on understanding how these drugs affect global cellular activity.

**Methods:**

Using FTase inhibitor I and genome-wide chemical profiling of the yeast barcoded deletion strain collection, we identified genes whose inactivation increases the antiproliferative action of this FTI peptidomimetic. The main findings were validated in a panel of cancer cell lines using FTI-277 in proliferation and biochemical assays paralleled by multiparametric image-based analyses.

**Results:**

ABC transporter Pdr10 or p-21 activated kinase (PAK) gene deletion increases the antiproliferative action of FTase inhibitor I in yeast cells. Consistent with this, enhanced inhibition of cell proliferation by combining group I PAK inhibition, using IPA3, with FTI-277 was observed in melanoma (A375MM), lung (A549) and colon (HT29), but not in epithelial (HeLa) or breast (MCF7), cancer cell lines. Both HeLa and A375MM cells show changes in the nuclear localization of group 1 PAKs in response to FTI-277, but up-regulation of PAK protein levels is observed only in HeLa cells.

**Conclusions:**

Our data support the view that group I PAKs are part of a pro-survival pathway activated by FTI treatment, and group I PAK inactivation potentiates the anti-proliferative action of FTIs in yeast as well as in cancer cells. These findings open new perspectives for the use of FTIs in combinatorial strategies with PAK inhibitors in melanoma, lung and colon malignancy.

## Background

Farnesyltransferase inhibitors (FTIs) are broad-spectrum low-toxicity anticancer agents originally isolated from fungi to inhibit Ras oncoprotein membrane attachment and therefore their malignant transforming activity [1,2]. The FTI Manumycin A was the first to be selected using a yeast-based genetic screen [3,4]. More than two decades of studies, using structurally different FTI compounds tested on several tumor cell lines, xenograph and cancer animal models, have confirmed that they act via evolutionarily-conserved mechanisms by inhibiting farnesyltransferase activity [1,2,5-7]. Surprisingly, FTIs were found to be effective also in Ras-independent tumors. Despite several studies, how FTIs act as anti-replicative compounds remains to be fully elucidated: hundreds of proteins are farnesylated in human cells, among which are several proteins activating pro-survival pathways. Inhibition of farnesylated proteins such as RheB or CENP-E appears to be among the consolidated data for some non-Ras tumors sensitive to FTIs. Complicating this picture, recent data suggest that farnesylation-independent pathways might also participate in the anticancer activity of FTIs [8-10].

Despite this lack of knowledge, the low toxicity of FTIs for normal cells and their wide-range of high anti-proliferative action on tumor cells led to the introduction of orally-available FTI molecules into clinical trials [5,6,11]. The FTI Tipifarnib (Zarnestra, R115777) has been evaluated for the treatment of myeloid malignancy, including for elderly patients with acute myelogenous leukemia (AML) [6,12]. Moreover, Tipifarnib has shown promising results in coadjutant therapies for breast cancer [13]. The FTI Lonafarnib have shown efficacy in melanoma cells that develop resistance to Sorafenib, a pan-Raf inhibitor [14]. The poor performance of FTIs at the clinical level compared to their anticipated wide use in anticancer therapy clearly shows the weakness of the mechanistic studies performed thus far. The further exploitation and future introduction of FTIs into clinical therapy will largely depend on the identification of compounds that increase FTI antiproliferative action in resistant tumors and on the identification of susceptibility prediction markers [5,6,11].

The major limitation of proteomic approaches undertaken thus far devoted to clarifying which farnesylated proteins are differentially prenylated upon FTI treatment has been the difficulty of correlating the effective protein prenylation status with their anti-proliferative action [5,6]. Several types of genomic technologies have been used to identify predictive markers/pathways that could explain how FTIs affect cellular activity and responsiveness. A handful of genes has been identified whose function might lead to FTI resistance [6,15-17]. Lack of FTI responsiveness has been shown to result from innate or acquired resistance or from FTI-mediated activation of pro-survival pathways. In addition, mutation of FTase or target genes, activation of alternative prenylation pathways, or changes in the balance of prenylated proteins have been described extensively upon FTI treatment [5,6,11].

To identify the major protein networks responding to FTI peptidomimetics as well as the major pathways that allow an escape from the anti-proliferative action of FTIs in yeast and mammalian tumor cell lines, we used budding yeast cell-based “omic” approaches and then validated the main findings in mammalian cancer cell lines. Well-characterized structurally related FTI compounds that are active in yeast or in mammalian cells, FTase inhibitor I and FTI-277, respectively, were used in order to compare the data. We expect that the basic knowledge obtained by these studies will give a better view of how to use clinically useful FTIs in combinatorial therapies. With this long-term goal in mind, in a previous study we profiled gene expression upon FTase inhibitor I treatment of yeast cells. Transcriptional and localization changes of P-glycoproteins belonging to the ABC transporter family acting in sphingolipid metabolism and drug resistance were observed [10]. Other transcriptional changes were found for genes encoding proteins that act in key signal transduction pathways regulating cell cycle entry and chromosome segregation and nutritional cues. We showed that these effects were specific to FTase inhibitor I (not being related to GGTase I inhibition or FTase subunit gene deletion in yeast cells). Multiparametric functional studies were carried out in HeLa cells to validate these observations. Nuclear morphology, Aurora A localization and S6 phosphorylation were found to be affected by FTI-277 treatment of HeLa cells [10]. Collectively these findings showed that FTIs have several unexpected effects on signaling pathways regulating proliferation that are not directly related to farnesylation and that these effects could be reciprocated in HeLa cells.

To identify genes whose deletion increases the anti-proliferative action of FTI peptidomimetics, here we report the chemical-genetic profiling of the yeast *Saccharomyces cerevisiae* barcoded deletion strain collection using FTase inhibitor I. Two p-21 activated kinases (PAKs), Cla4 and SKM1, and the ABC transporter Pdr10 were among the genes whose deletion increased FTI sensitivity in yeast cells. To test whether PAK inhibition might increase FTI sensitivity in cancer cell lines resistant to FTIs, we measured the proliferation of HeLa, melanoma (A375MM), lung (A549), colon (HT29) and breast (MCF7) cancer cell lines after FTI-277 treatment, administrated alone or in combination with a highly selective group I PAK inhibitor, named IPA3 [18,19]. We show that the use of IPA3 at concentrations ranging from 5 to 7 μM in combination with 5 μM FTI-277 potently inhibits proliferation of A375MM melanoma, A549 lung and HT29 colon cancer cell lines, but hardly affects the proliferation of HeLa or MCF7 breast cancer cell lines.

## Results

### The ABC transporter Pdr10 and p-21 activated kinases act in pro-survival pathways mediating FTI peptidomimetic susceptibility in yeast cells

To identify genes promoting survival to FTI peptidomimetic treatment in eukaryotic cells, we performed a genome-wide drug sensitivity screen using the barcoded yeast deletion mutant collection (representing approximately 4700 genes) and 10 μM of the peptidomimetic FTase inhibitor I (Calbiochem-MERK). We have shown previously that 10 μM FTase inhibitor I treatment of BY4741 cells induces specific changes in the yeast transcriptome without affecting Ras binding to the plasma membrane [10].

**Figure 1:**
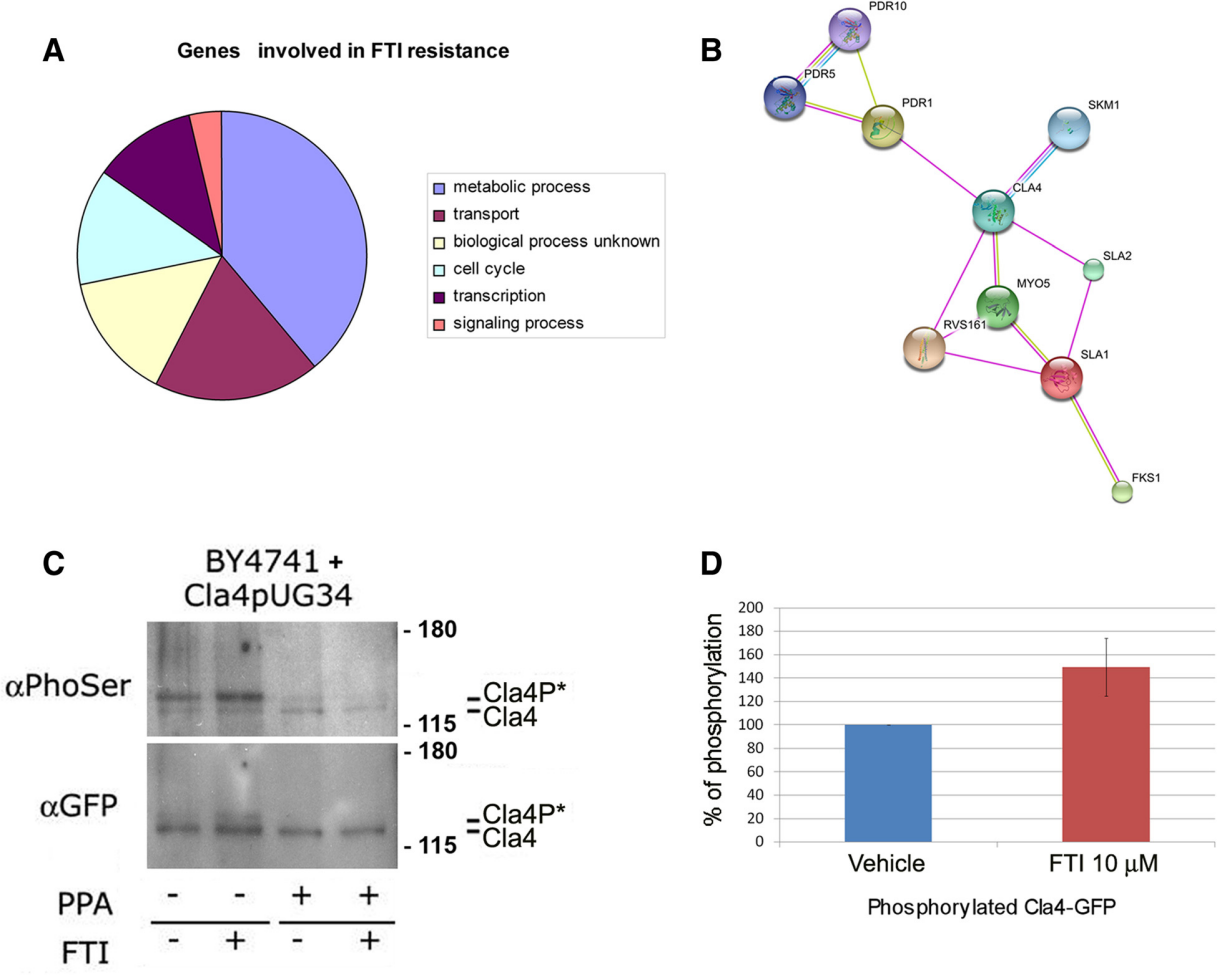
**p-21 activated kinases or ABC transporters are among the genes whose deletion increases FTI sensitivity of yeast cells. A**. Pie visualization of the 64 genes identified by drug sensitivity screening as putative hits. Super GO Slim tool Biological Process Binning was used for clustering the FTI hypersensitive hits. **B**. The network analysis of hypersensitive strains carrying deletions of CLA4, SKM1, PDR10 was performed for known and predicted protein-protein interactions using STRING (http://string-db.org/). The evidence view is shown, different line colors represent the types of evidence for the association. High confidence (score 0.700) excluding the text mining settings were used as parameters. **C**. Immunoblot analysis of the immunoprecipitates obtained from BY4741 cells expressing GFP-Cla4. αPhoSer = anti-phosphoserine Q5 antibody; αGFP = anti-GFP antibody. (+) indicates addition, (−) no addition. PPA = phosphatase lambda. FTI = 10 μM FTase I inhibitor. Numbers indicate MW. Expected protein positions are indicated. **D**. The graph shows the relative amount of phosphorylated versus unphosphorylated Cla4 relative to vehicle. The relative protein levels are expressed in percentage (%). The amount of control (vehicle-treated cells) was considered as 100%. Error bars indicate means ± SD of at least 3 replicates (n = 3).

The genome-wide sensitivity screen highlighted sixty-four genes whose deletion results in a two-fold increase in FTI sensitivity (log2 ratio > 0.5, p-value < 0,05; Additional file 1: Table S1). These sixty-four hits were further classified according to Gene Ontology criteria using the Super GO-Slim Process clustering tool available at the GO-SGD database (http://www.yeastgenome.org). This analysis showed that 25% of the genes promoting survival to FTI peptidomimetic treatment act in transport and 15.6% are annotated as being involved in cell cycle processes (Figure 1A; Additional file 2: Table S2). The functional associations among the hits involved in transport were further analysed using STRING (version 8.3, http://string.embl.de/). This analysis showed that Pdr10, an ATP-binding-cassette (ABC) transporter belonging to the multidrug resistant (MDR) gene class, and the PAKs CLA4 and SKM1 form a gene network with the ABC transporter PDR5 and the PDR transcriptional regulator PDR1 (Figure 1B).

We showed previously that PDR5 and PDR1 are transcriptionally up-regulated and that Pdr5 recycling increases in FTase inhibitor I-treated yeast cells [10]. Moreover, Pdr5 recycling depends on END4 [20], which interacts with the PAK Cla4p [21], suggesting the existence of a functional network that connects PDR5 recycling at the plasma membrane and PDR1 transcriptional up-regulation upon FTI drug treatment with increased sensitivity in the presence of a CLA4 or PDR10 gene deletion.

To test this idea, we determined the levels of Cla4p and its state of phosphorylation in yeast cells expressing a GFP-tagged version of Cla4 (GFP-Cla4) treated with FTase inhibitor I. GFP-Cla4 localizes like the wt protein when expressed in BY4741 cells (Additional file 3: Figure S1). Total lysates prepared from GFP-CLA4-transformed cells treated with FTase inhibitor I (Figure 1C, lanes FTI +) or left untreated (Figure 1C, lanes FTI -) were immunoprecipitated (IP) using an anti-GFP antibody (αGFP) followed by immunoblot analysis. Total lysates were prepared in the presence (Figure 1C, lanes PPA +) or absence of λ-phosphatase (Figure 1C, lanes PPA –). After normalization against the total amount of Cla4p present in each sample, the amount of phosphorylated Cla4p was calculated (Figure 1D). An average (n = 3) increase of 50% in phosphorylated Cla4p was observed in FTase inhibitor I-treated samples (Figure 1C, lanes FTI + PPA -) compared to controls (Figure 1C, lanes FTI – PPA -).

Thus, we concluded that FTase inhibitor I treatment promotes activation of the PAK kinase Cla4p in yeast cells.

### FTI-277 promotes group I PAK expression in HeLa but not in A375MM cells

PAK kinases are serine/threonine protein kinases that are activated in response to various signalling pathways that regulate proliferation, cell shape and motility in mammalian cells. PAK protein levels have been correlated with proliferation in several human tumors and are known to participate in metastatic processes [22,23]. However, how PAK function relates to FTI efficacy has never been investigated. Human PAKs can be subdivided into two main classes based on their structural characteristics. The current classification separates the yeast PAKs (Cla4, Ste20 and Skm1) from both mammalian PAK classes. However, based on complementation studies performed with PAK family members expressed in *ste20* mutants, the yeast PAKs are considered to be functionally related to group I PAKs [23-25]. Therefore, to determine the effects of FTI on PAKs in tumor cells we first assayed the levels of group I PAKs in HeLa and A375MM melanoma cell lines. HeLa and A375MM were used in these studies as prototypical cancer cell lines with different genotypes (Table 1) [26].

**Table 1:** Panel of tested tumor cell lines

**Cell line**	**Tumor type**	**Genotype**	**FTI sensitivity**	**Source**	**References**
HeLa	Cervical	p53 not expressed	Resistant	ECACC	[27]
A375MM	Melanoma	BRaf^V600E^	Resistant	[28,29]	[28-30]
HT29	Colon	BRaf^V600E^	Sensitive	ATCC	[26,31,32]
		p53^R273H^			
A549	Lung	KRas^G12S^	Sensitive	ATCC	[33]
MCF7	Breast	PI3KCA^E545K^	Sensitive	ATCC	[34]

Note: wt genes are not indicated.

We first measured the basal levels and phosphorylation of group I PAKs and their cytosolic/nuclear distribution in these cell lines upon FTI-277 treatment by automated fluorescence microscopy-based high-content phenotypic profiling using the acquisition and analysis platform of the microscopy station Scan^R^ (OLYMPUS). In these series of experiments the group I PAK and phosphorylated PAK protein levels were evaluated based on the fluorescence intensity using anti-PAK-C19 (αPak) or anti-phosphorylated PAK 1/2/3 [^Thr423^] (αPhoPak) primary antibodies and appropriately fluorescently-conjugated secondary antibodies, as previously described [10]. These experiments were paralleled by immunoblot analysis for independent validation. We chose to analyse the cells 4 h and 48 h after FTI treatment because these time points could be paralleled by proliferation studies.

**Figure 2:**
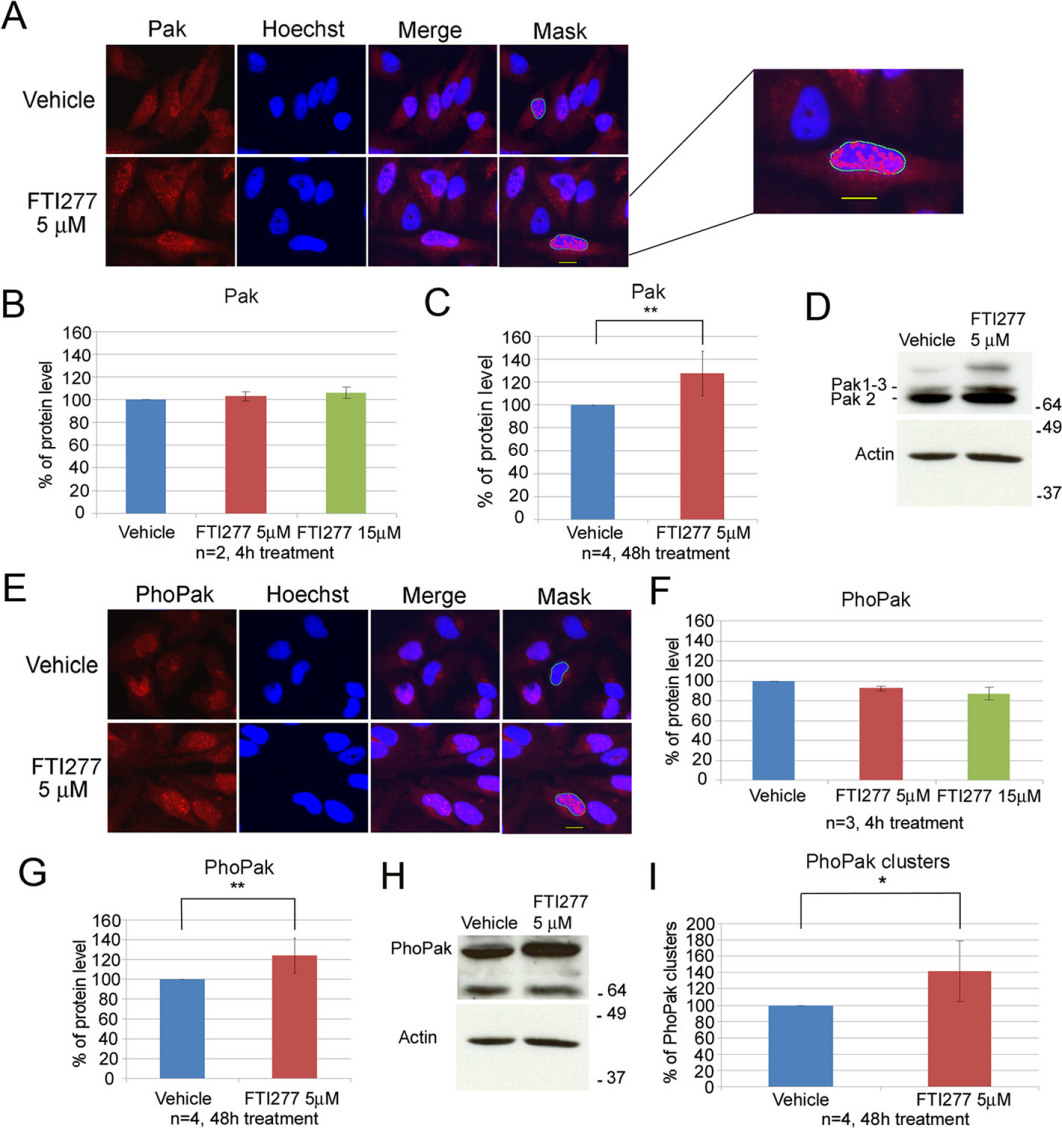
**FTI-277 treatment of HeLa cells up-regulates PAKs and phosphorylated PAKs.** The statistical significance of the treatments was calculated using t-test: ns = not significant, p-value >0.05; * = significant, p-value <0.05; ** highly significant = p-value <0.01). n = n. of biological replicates. Each biological replicate consider the average mean intensity data obtained from analysis of three wells per condition. **A**. Fluorescently stained HeLa cells treated for 48 h with the indicated concentration of FTI-277 or DMSO (Vehicle). Panels: PAK = anti-PAK (C19) antibody; Hoechst = nuclear staining; Merge = Hoechst + PAK; Mask: software mask that identifies nuclear PAKs (green), and PAK clusters (spots) within nuclei (red). scale bar =10 μm. **B**. The graph shows the relative amount (%) of PAKs in the nuclei in 4 h FTI277-treated cells relative to vehicle, arbitrarily set as 100%. **C**. The graph shows the relative amount (%) of total PAKs in 48 h FTI-277-treated cells compared to vehicle-treated cells; **D**. Immunoblot analysis of total lysates from HeLa cells treated as indicated in **C**. **E**. Fluorescently stained HeLa cells treated for 48 h with the indicated concentration of FTI-277 or DMSO (vehicle). Panels: PhoPak = α-Phospho-PAK1/2/3[pThr423] antibody; Hoechst = nuclear staining; Merge = Hoechst + PhoPak; Mask: software mask that identifies nuclear PhoPaks (green), and PhoPak clusters (spots) within nuclei (red). **F**. The graph shows the relative amount (%) of total PhoPaks in 4 h FTI277-treated cells relative to vehicle-treated cells. **G**. The graph shows the relative amount of total PhoPaks in 48 h FTI277-treated cells compared to vehicle-treated cells; **H**: immunoblot analysis of total lysates form HeLa cells treated as in **G**. **I**. The graph shows the relative number (%) of PhoPaks spots within the nuclei in 48 h FTI277-treated cells relative to vehicle-treated cells, which were arbitrarily considered 100%.

Image analysis showed that group I PAKs and their phosphorylated forms, hereafter named PAKs and PhoPAKs, respectively, localize in the cytoplasm as well as in the nucleus of HeLa cells (Figure 2A and E, respectively), as previously described [35-37]. PAKs and PhoPAKs cluster in spots of different dimensions in the nucleus (Figure 2A and E, respectively). After 4 h treatment with 5 μM or 15 μM FTI-277, this localization did not change substantially, nor were PAK protein levels affected (Figure 2B) although a slight decrease in the PhoPAK signal was observed (Figure 2F). By contrast, after 48 h of 5 μM FTI-277 treatment, a significant (p ≤ 0.01 n = 4) increase in the PAK (Figure 2C) and PhoPAK signal (Figure 2G; p ≤ 0.01 n = 4) was observed. Immunoblot analysis of samples treated in parallel experiments confirmed these trends (Figure 2D and H, respectively). Moreover, a significant increase (p ≤ 0.05) in PhoPAK clusters within the nuclei was observed (Figure 2I).

**Figure 3:**
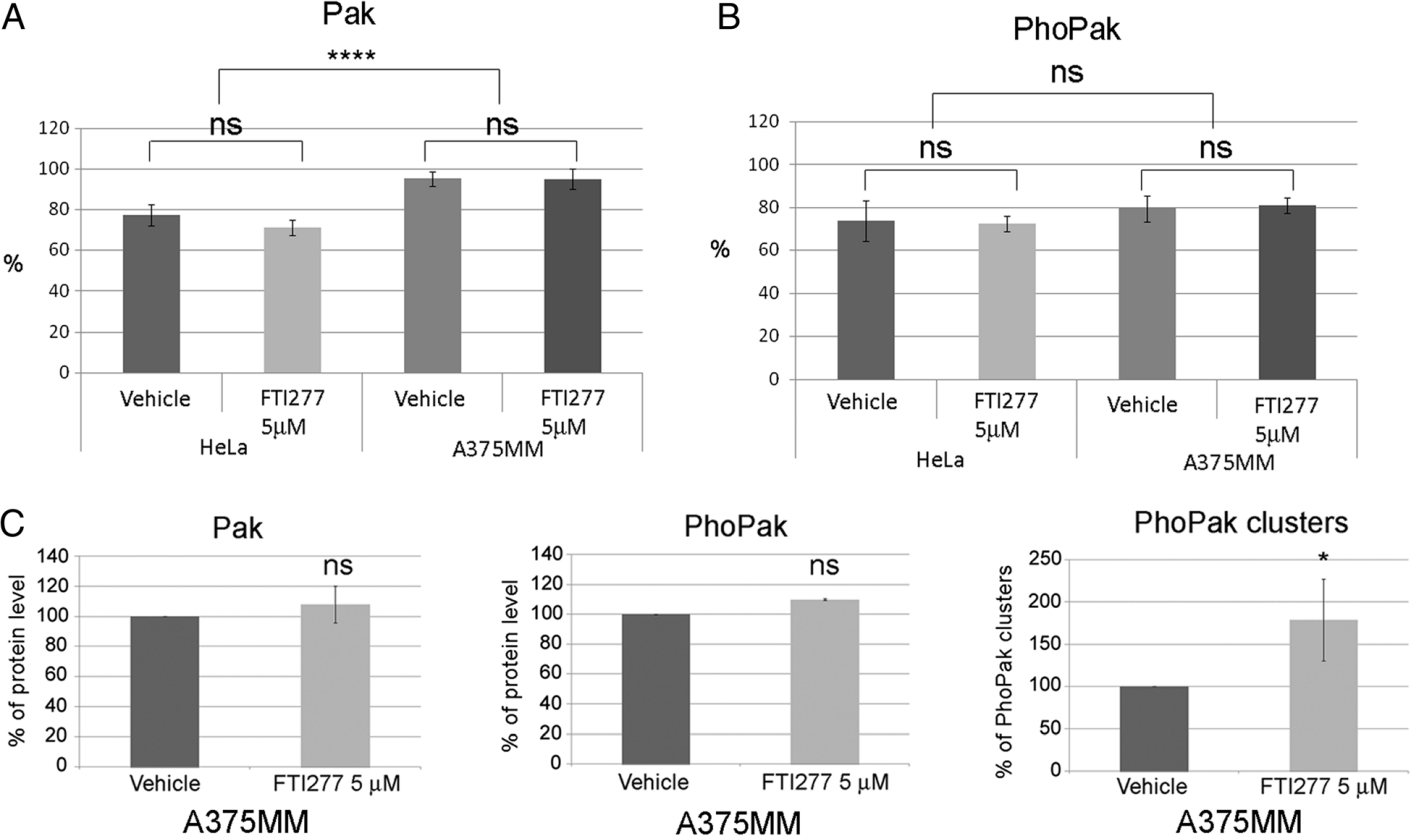
**FTI-277 does not affect the localization of PAKs and PhoPAKs in HeLa and A375MM cells.** HeLa and A375 MM cells were treated for 48 h as indicated in Figure 2 and in Methods, and stained with relevant antibodies. Olympus Scan^R^ analysis software was used to calculate the intensity of the fluorescent signal present in nuclei and in the whole cells. **A**. The graph represents the relative amount (%) of PAKs present in nucleus versus the amount present in the whole cell in the indicated treatment. **B**. The graph represents the relative amount (%) of PhoPAKs (respective panels) present in nucleus versus the amount present in the whole cell in the indicated treatment. **C**. The graph represents the relative amount of PAKs, PhoPAKs, and PhoPAK clusters in A375MM cells treated for 48 h as indicated in Figure 2 and in Methods. The mean intensity of the signal in vehicle samples was arbitrarily considered 100%. The amount of PhoPAK spots present in the nuclei of each sample was normalized against the total number of cells counted. All graphs (panel **A**, **B**, **C**) shows the mean ± SD of at least 2 independent experiments, each run in triplicate (three wells per condition). The statistical significance of the indicated treatments was calculated using t-test: ns = not significant, p-value >0.05; * significant, p-value < 0.05; **** = highest significance, p-value <0.0001.

We further compared the PAK and PhoPAK localization in HeLa and A375MM cell lines treated and untreated with FTI-277. We observed that PAK localization differs significantly in these cell lines. In A375MM melanoma cells, 95% of PAK proteins reside within the nuclei, while in HeLa cells only 77% of the protein shows this localization (Figure 3A). Upon FTI-277 treatment we failed to observe any effect on PAK protein levels in A375MM melanoma cells. However, as in HeLa cells, the PhoPAK clusters within the nuclei increase significantly over control (Figure 3C panel PhoPak clusters). These data indicate that although the majority of PAK resides within the nuclei in A375MM cells, FTI-277 treatment causes a change from a diffuse to a clustered state of this protein (Figure 3C) but does not affect the overall amount of PAK protein, as occurs in HeLa cells.

To further investigate how FTI-277 treatment affects PAK activity in HeLa cells, we investigated the cell adhesion capabilities of treated versus control cells. It is well established that the interaction of PAKs with the cytosolic PIX-GIT/Paxillin signaling module increases cell motility by promoting focal adhesion (FA) turnover and disassembly [23,25,38]. A way to estimate FA assembly is to estimate the amount of vinculin at membranes, as vinculin reduction correlates with reduced FA formation and increased cell migration rates [24,39,40]. Thus, we determined the effects of FTI-277 on cell adhesion by following vinculin recruitment to FAs in HeLa cells, treated with 5 μM or 15 μM FTI-277 or with vehicle using automated fluorescence microscopy on cells plated in 96-well plates, fixed and processed for image analyses as described above.

**Figure 4:**
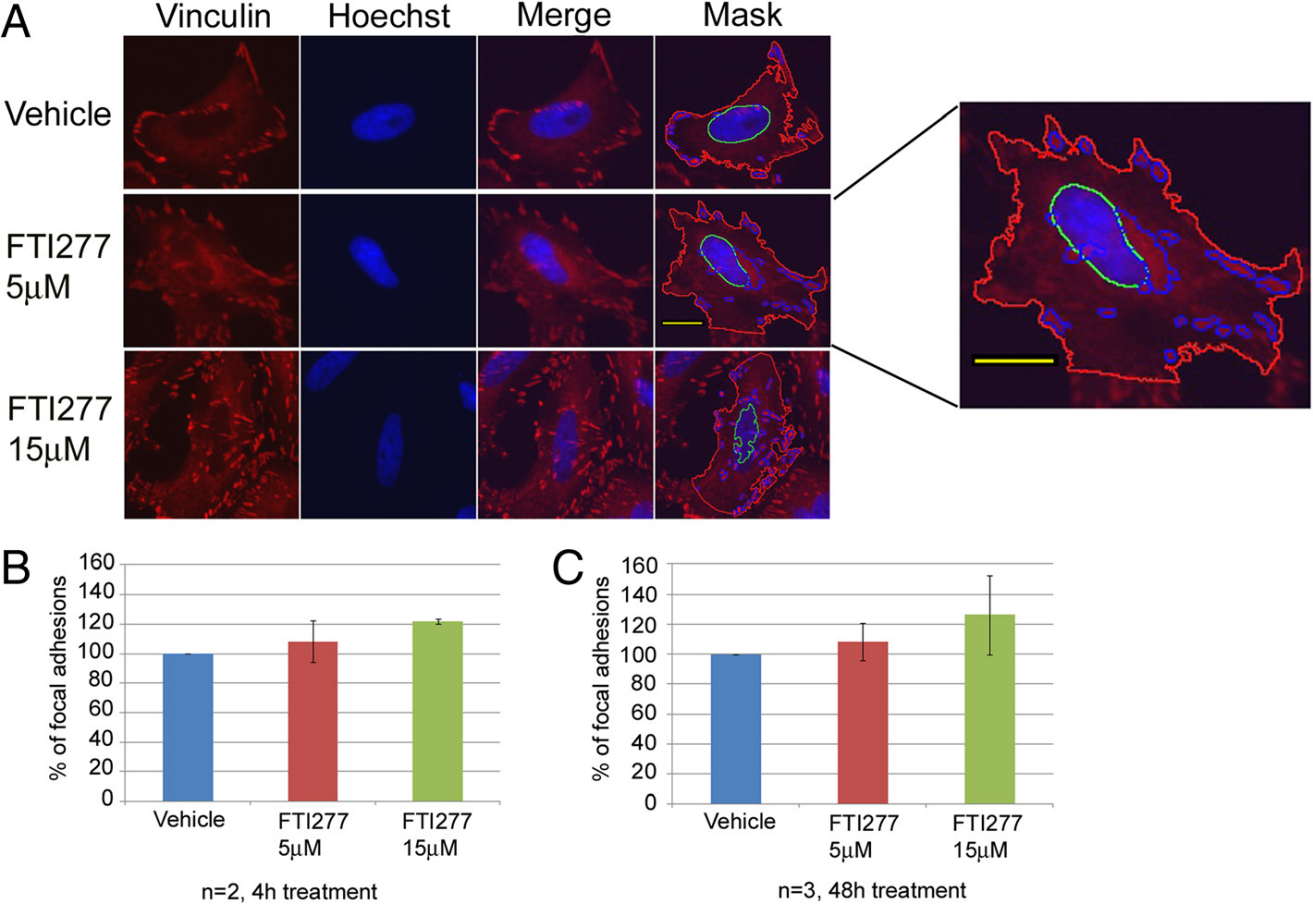
**FTI-277 treatment of HeLa cells increases focal adhesions. A**: The images show the localization of vinculin in FTI277-treated and vehicle-treated samples together with the software mask to identify total vinculin (red) and vinculin at the focal adhesions (blue). HeLa cells were treated as indicated in the text and stained for immunofluorescence analysis with α-vinculin primary and appropriate secondary fluorescently labeled antibody, as indicated in Methods. To calculate the number of focal adhesions in each experiment at least 86 cells were counted per sample. The software mask corresponding to the vinculin signal is highlighted (panel A, Mask, blue circled spots). The number of focal adhesions present in each sample was counted and normalized against the total number of cells counted. The number of focal adhesions in vehicle samples was arbitrarily considered 100%. The yellow scale bar represents 10 μM. **B** and **C**. The graphs show the mean ± SD of at least 2 independent experiments, each run in triplicate (three wells per condition) at the indicated time points.

As expected, in vehicle-treated samples, vinculin clusters at the membrane were observed, indicating FA formation (Figure 4A panel Vehicle) [39]. Treatment with 5 μM or with 15 μM FTI-277 for 4 h resulted in an increased number of FAs containing vinculin compared to control samples (Figure 4B). The time of treatment did not substantially affect this trend (Figure 4C). These data indicate that although the overall PAK levels in HeLa cells increase (Figure 2C and G), there are no affects of the cytosolic PAK activity on FAs.

### Combining the PAK inhibitor IPA3 with FTI-277 exerts a potent antiproliferative action in melanoma, lung and colon cell lines

The large number of possible group I PAK activators in proliferating cells [22], many of which remain unknown [23], makes it difficult to identify proteins that might activate group I PAKs in the nuclei of different cancer cell lines. Therefore, we first focused on determining the effects of PAK inhibitors on the panel of cancer cell lines listed in Table 1 using MTS-based proliferation assays. MCF7 breast cancer, HT29 colon cell line and A549 lung cancer cell line are reported to be FTI-sensitive cell line [19,26,41], while HeLa cervical and A375MM melanoma cell line are reported to be resistant to FTIs [14,26]. The PAK inhibitor IPA3, which targets the Cdc42-mediated autophosphorylation of threonine 423 in group I PAK proteins, was used in these studies as it is highly specific [42].

Proliferation tests were performed using a range of concentrations of IPA3 previously shown to affect the proliferation of different tumor cell lines [43]. In preliminary tests we also determined the toxic concentration of IPA3 in HeLa cells and A375MM cells. We observed that although HeLa cells are fairly resistant to this compound, 48 h treatment with 20 μM IPA3 is toxic for this cell line (Additional file 4: Figure S2). Based on this, a concentration of 2, 5, or 7 μM IPA3 was use in further studies. To perform these experiments, HeLa, A375MM, HT29, A549 and MCF7 cancer cell lines were left to attach for 24 h in 96-well plates, treated with 5 μM FTI-277 or with 2, 5, or 7 μM IPA3 administrated alone, or with a combination of FTI-277 and IPA3. The cells were then incubated for a further 48 h prior to data acquisition as described in Methods.

**Figure 5:**
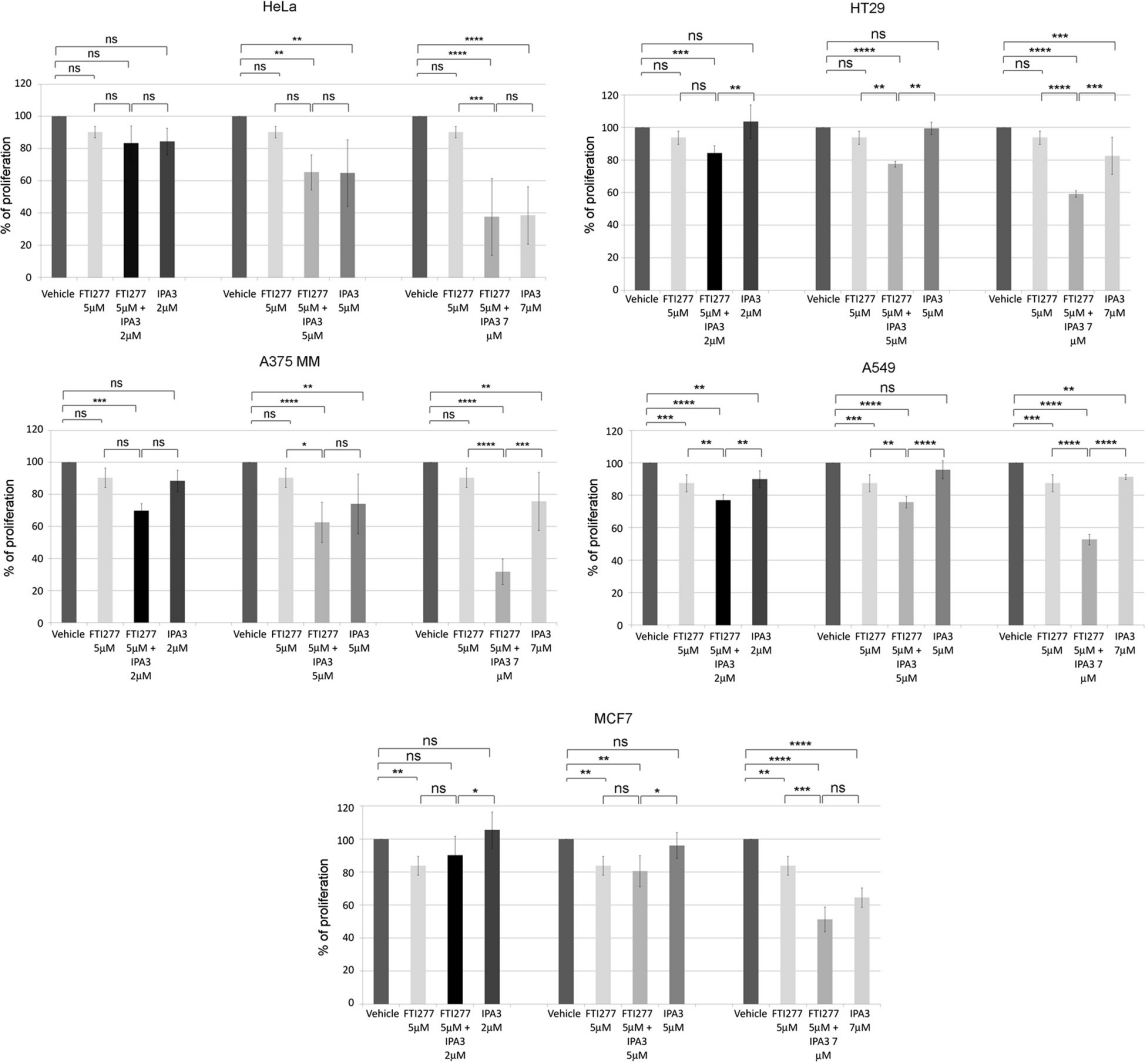
**Combined use of FTI-277 and PAK inhibitor IPA3 reduce proliferation of A375MM, HT29, A549 cells but not of HeLa or MCF7 cells.** Effects of FTI-277 and/or IPA3 on cell proliferation in the indicated cells.% is relative to the vehicle that was considered as 100%. Error bars indicate means ± SD of 3 independent experiments from 5 wells. The statistical significance of the treatments was calculated using t-test as described in Methods: ns = not significant, p-value >0.05; * = significant, p-value <0.05; ** highly significant = p-value <0.01; *** very highly significant = p-value < 0.001; **** highest significance = p-value < 0.0001.

We observed that A549 cells and MCF7 cells were sensitive to 5 μM FTI-277 (Figure 5), while the other cell lines were not. All cell lines were sensitive to 7 μM IPA3, HeLa and MCF7 cells being the most sensitive, while A549, A375MM and HT29 cells show only moderate sensitivity (Figure 5, respective panels). The combined use of 7 μM IPA3 and 5 μM FTI-277 resulted in the strongest inhibition of proliferation (p-value < 0.0001) in all cell lines, A375MM cells being the most sensitive. However, it should be noted that the combination of 5 μM FTI-277 and 7 μM IPA3 did not substantially change the basal sensitivity of HeLa and MCF7 cells observed using 7 μM IPA3 alone.

We concluded that inhibition of group I PAKs using IPA3 combined to FTI-277 treatment potently inhibits the proliferation of A375MM, A549 and HT29 cancer cell lines, while IPA3 is highly effective in inhibiting the proliferation of HeLa and MCF7 cancer cell lines independently of FTI treatment (Figure 5).

To determine if the different proliferative ability of HeLa compared to A375MM cells in the presence of 5 μM FTI-277 and 7 μM IPA3 was due to an increase in the number of apoptotic cells, we analyzed the percentage of cells that had fragmented nuclei using the Scan^R^ analysis software [10]. FTI-277 treatment of A375MM cells led to a significant increase in the number of apoptotic cells, which was reduced when the cells were co-treated with IPA3, suggesting that IPA3 has a protective effect against apoptosis (Additional file 5: Figure S3, respective panels). These data indicate that IPA3 counteracts the pro-apoptotic activity of FTI-277 in this cell line. By contrast, no major effects were observed on HeLa cells using either drug alone or in combination (Additional file 5: Figure S3, respective panels).

**Figure 6:**
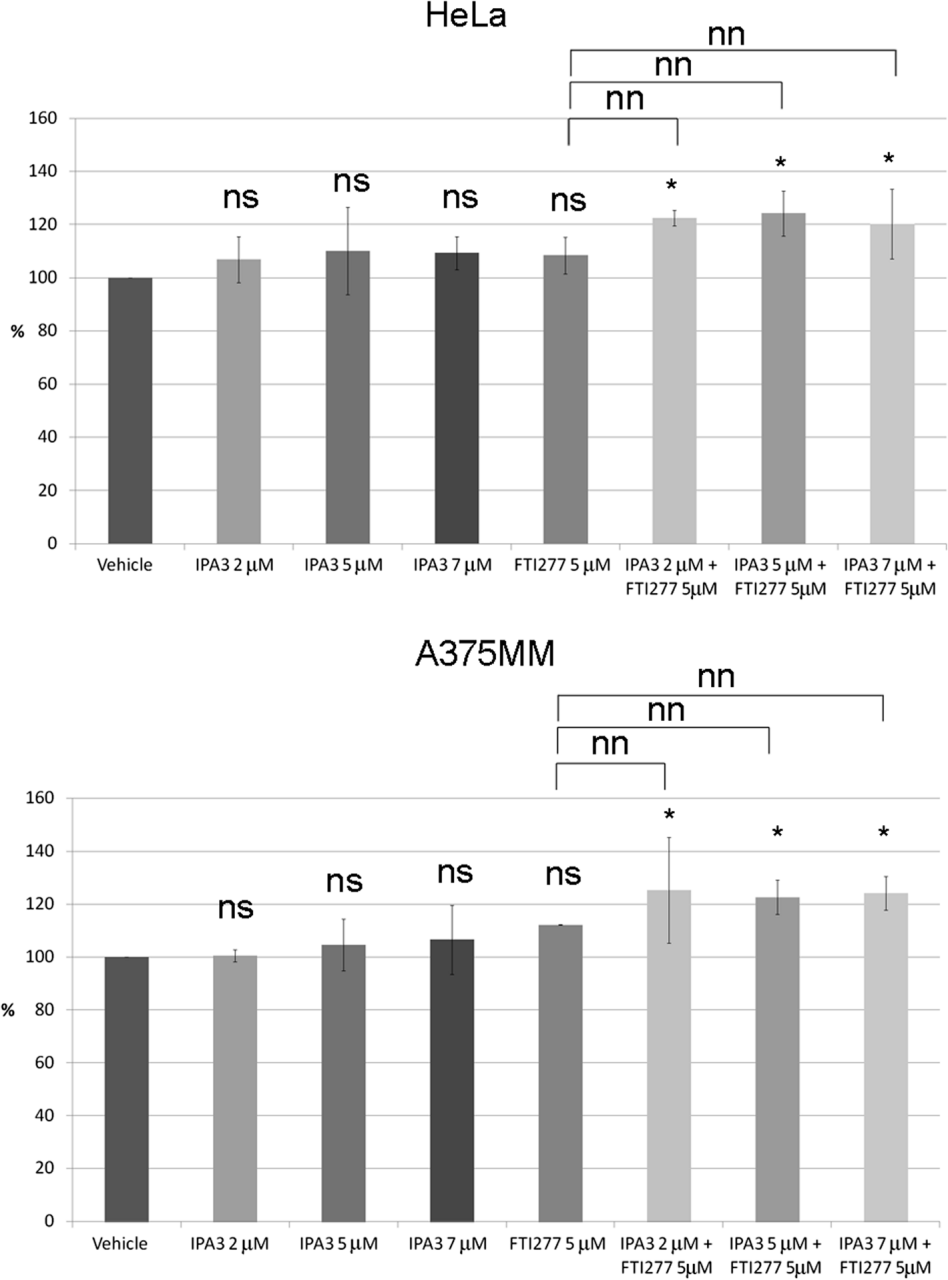
**Combined treatment of FTI-277 and IPA3, but not FTI-277 alone, increases the cell size of HeLa and A375MM cells.** HeLa and A375MM cells were treated for 48 h as indicated in Figures 2, 3 and in Methods, and stained with relevant antibodies. Olympus Scan^R^ analysis software was used to calculate the area of each cell counted. The graph represents the relative area (%) of cells treated as indicated for HeLa and A375MM cells (respective panels) with cells treated with vehicle, arbitrarily set at 100%. The graph shows the mean ± SD of 2 independent experiments, each run in triplicate (three well per condition). More than 73 cells for A375MM and 573 cells for HeLa cells were counted per sample in each experiment. Results of t-test are shown above the graph: ns: no significant deviation from vehicle, p-value >0.05; * p-value <0.05; nn: no significant deviation from FTI-277, p-value >0.05.

To estimate the number of senescent cells, we measured the mean area of cells compared to control [44] after FTI-277 treatment in the presence or absence of different concentrations of IPA3 using the Scan^R^ analysis software. We observed that the combined treatment of FTI-277 and IPA3 resulted in a statistically significant increase in the overall cellular area in both HeLa and A375MM cells compared to vehicle-treated cells but not compared to FTI-277 treated cells (Figure 6).

## Discussion

Group 1 PAKs are key players in cellular mechanisms that are important for transformation, tumor progression and metastatic processes [23]. Here we show that the combined use of group I PAK inhibitors and FTI-277 exerts a potent anti-proliferative action in melanoma, colon and lung cancer cell lines. Given the refractory to conventional treatments of these tumors, these findings open the possibility of using FTIs in combinatorial therapies with PAK inhibitors for these aggressive tumors. Importantly, our data show also that the underlying mechanism of how PAK down-regulation and FTIs exerts an anti-proliferative action on eukaryotic cells is evolutionarily conserved.

Our genome-wide FTI sensitivity screen data indicate that deleting the ABC transporter gene PDR10 is one way to increase FTI sensitivity in yeast cells. ABC transporters constitute a large family of proteins that act as detoxification pumps in yeast as well as in mammalian cells [45]. They are known to participate in drug resistance in various ways and to be up-regulated in several tumors [20,45,46]. The data reported here support previous yeast genome-wide expression profiling studies showing that the ABC transporter Pdr5 and its transcriptional regulator Pdr1 respond to FTI drug intake in yeast cells by up-regulating their activity [10]. Importantly it has previously shown that Pdr5 recycling from the plasma membrane to endosomes depends on END4/SLA1, which interacts directly with the PAK kinase Cla4 [20,21]. Recent epistasis studies indicate that Pdr10 has a complementary function with Pdr5. Moreover, Pdr10 function depends on Pdr5, Pdr12, Lem3 and sphingolipids [47-49]. Taken together these data, our expression and chemical profiling of yeast cells treated with FTI inhibitor I, it can be envisaged that it exists a functional network that connects FTI uptake at the plasma membrane by ABC transporters acting in sphingolipid metabolism and PAK activation. Consistent with this, we showed previously that FTase inhibitor I promotes Pdr5 recycling from the plasma membrane [10]. The existence of a functional network that connects FTI uptake, ABC transporter recycling and PAK activity is also supported by the phenotypic analysis of yeast cells lacking of the PAK CLA4 (∆*cla4*)*:* a drastic reduction in drug resistance and in the transcription of the ABC transporter PDR5 was shown [47,50]. Here we show that the PAK Cla4 is activated in FTase inhibitor I-treated yeast cells. A role for some classes of the ABC transporter family in FTI resistance in mammalian tumors has been previously suggested by genome-wide expression profiling studies performed with the FTI Tipifarnib [8,9,17]. However, the large number of ABC transporters encoded by the human genome, their different distribution in different cancer cell lines, and their redundant functions [45], makes it difficult to identify which of them might be specifically involved in FTI uptake in the tumors studied in this study.

The data obtained here indicate that in the presence of FTI-277, PAKs sustain proliferation of melanoma, colon and lung cancer cell lines, but unlikely of HeLa or MCF7 cell lines. Proliferation inhibition caused by the combined use of FTI-277 and IPA3 ranged from 40% for HT29 cells to 68% for A375MM cells. In case of HT29 and A549 cells, even the lowest concentration (2 μM) of IPA3 used significantly inhibited proliferation when combined FTI-277 compared to IPA3 alone. The poor response of HeLa and MCF7 tumor cell lines to the combined use of the PAK inhibitor and FTI-277, compared to the significant responsiveness of A375MM, HT29 and A549 cell lines, must reside in the mechanism that determines how FTI peptidomimetics act as anti-proliferative drugs in these cell lines. A375MM and HT29 are mutated in BRafV600 and A549 carry a K-Ras mutation while HeLa cells have wt Ras. It is known that A375MM cells rely on the activation of two main signaling pathways that sustain proliferation: RAF-MEK-ERK (MAPK) and PI3K-AKT-mTOR (AKT) signalling. The FTI inhibitor Lonafarnib acts through inhibition of mTOR signaling independently of MAPK or AKT activation [14]. Given these data it is conceivable that FTI-mediated PAK activation acts in synergy with MAPK and AKT pathways or is part of these pathways in these cell lines. Thus not only the mechanism by which PAK down-regulation exerts an anti-proliferative action in the presence of FTIs must be a basic and well-conserved process in the evolution, but also these data show that the combined use of FTIs and PAK inhibitors potently act as antiproliferative drugs in unrelated aggressive cancers characterized by constitutive activation of MAPK and AKT pathways. This latter view is also supported by the recently reported function of PAK1 in stimulation of colorectal proliferation by gastrins via multiple signalling pathways involving activation of ERK, AKT, and β-catenin [51]. Increased p21-activated kinase-1 expression is associated with invasive potential in uveal melanoma [52]. Thus, it is conceivable to think that the contemporaneous shut-down of either the RAF-MEK-ERK (MAPK) or PI3K-AKT signaling pathway might at the basis of the susceptibility of HT29, A375MM and A549 cells to FTI and PAK inhibitors.

All together our mammalian data substantially confirm the yeast data showing that PAK inhibition cooperates with FTIs in inhibiting proliferation of eukaryotic cells. The susceptibility of the A549 lung cancer cell line, which harbours a K-Ras mutation, to the combined use of IPA3 and FTI-277 is of particular interest, given the aggressiveness of current treatments for lung cancer. It has been previously shown that A549 cells treated with FTI-277 are blocked at the G2/M transition [19]. Interestingly, it was observed that antibodies developed against a specific C-terminal Ste20/PAK homologue facilitates the release of *Xenopus* oocytes from G2 arrest [53]. Given the observation that a combination of FTI-277 and IPA3 significantly increases the proportion of senescent A375MM cells, we propose that the combined effects of FTI-277 and PAK inhibitor IPA3 might similarly release A549 cells from the FTI-mediated G2/M block and promote senescence. To try to answer why the combinatorial use of IPA3 and FTI-277 does not reduce HeLa cell proliferation, we analysed the activation status and the intracellular localization of PAKs in HeLa and A375MM cell lines. However, none of the parameters measured correlated with the different effects that PAK inhibitors have on the respective proliferation abilities. In HeLa cells the effects of FTI-277 on FA assembly and vinculin recruitment are consistent with the anti-proliferative function of FTIs and with the view that cytosolic PAK/PIX/GIT module activation is not involved in the FTI-mediated PAK activation response.

## Conclusions

This work firmly establishes that PAK inactivation combined with FTI treatment has a potent anti-proliferative action on yeast as well as melanoma, colon and lung cancer cells. Further work will be required to elucidate how PAK inhibitors aid FTI anti-proliferative action in these tumor cell lines. Based on the yeast data [10], we suggest that ABC transporter recycling, consequent to FTI uptake, is the initial signal that activate PAK.

## Methods

### Yeast strains, plasmid constructs, media and growth conditions

Strains and oligonucleotides are listed in Tables 2 and 3, respectively. Media, yeast transformation and genetic manipulation as well as molecular procedures were as described previously [54]. Unless otherwise specified, yeast cells were grown at 28°C with agitation in YPD medium or in SD medium lacking the appropriate amino acid for plasmid selection as previously described [54]. To construct GFP-tagged Cla4, the Cla4 ORF was amplified by PCR from genomic DNA with the oligonucleotides listed in Table 3 using the High Fidelity Polymerase Chain Reaction kit (Roche). The PCR product was digested XmaI/EcoRI and ligated into the vector pUG34 as described previously [54].

**Table 2:** Strains

**Strains**	**Genotype**	**Source**
BY4741 wt	MATa, leu2, ura3, his3, met15	EUROSCARF
Deletion strain collection research genetics	MATa haploid deletion mutants	[55]

**Table 3:** Oligonucleotides

Cla4XmaI-Fw	AAATCCCGGGAATATGTCTCTTTCAGCTGCAGCGA
Cla4EcoRI-Rv	AATAGAATTCGATATGCTTATAGAAATAGTTGTG

### Reagents and antibodies

FTase inhibitor I (Cat.No 344510) and FTI-277 (Cat. No. 344555) were purchased from Merck-Calbiochem and were used according to the manufacture’s protocols as described in [10]. The p-21 activated kinase inhibitor IPA3 (1,1′-Disulfanediyldinaphthalen-2-ol, Cat.No. I2285) was purchased from Sigma. Antibodies are listed in Table 4.

**Table 4:** Antibody list

**Name**	**Manufacture/code**	**Protein target**
α-GFP	Roche, 11814460001	GFP
α-PhosphoSerine Antibody Q5	Qiagen, 37430	Serine-phosphorylated proteins
α-PAK (C19)	Santa Cruz, Sc881	PAK 1/2/3
α-Phospho-PAK1/2/3[pThr423]	Sigma, P7746	Phopshorylated PAK1/2/3 [pThr423]
α-Actin	Sigma, A2066	Actin
α-Vinculin, clone FB11	Chemicon, MAB1624	Vinculin
AlexaFluor 546 Goat α-Rabbit IgG	Invitrogen, A11035	Rabbit IgG
AlexaFluor 546 Goat α-Mouse IgG	Invitrogen, A11030	Mouse IgG
AlexaFluor 488 Goat α-Rabbit IgG	Invitrogen, A11034	Rabbit IgG
AlexaFluor 488 Chicken α-Mouse IgG	Invitrogen, A21200	Mouse IgG

### Yeast protein extraction, immunoprecipitation and immunoblot analysis

BY4741 cells carrying the plasmid GFP-Cla4 pUG34 were grown in the presence or absence of 10 μM FTase inhibitor I in selective media as previously described [10]. Typically, the drug was added to cultures diluted to an OD_600_ = 0.08 and the cells were harvested at OD_600_ = 0.6. To prepare crude extracts for phosphoprotein detection, the cells were diluted 1:1 in Stop Mix (0.9% NaCl, 1 mM NaN_3_ 10 mM EDTA, 50 mM NaF), washed once in Stop Mix, and resuspended in Lysis Buffer (50 mM Tris–HCl pH 7.5, 0.1 mM EDTA, 1 mM DTT, 1 mM PMSF) containing protease inhibitor and phosphatase inhibitor tablets (Roche) as described [56]. Crude extracts were obtained by the glass beads method and glycerol was added to a final concentration of 20%. The protein concentration was determined using the Bradford assay as described [54]. Immunoprecipitation and immunoblot analysis were performed as described previously [57]. Results were analysed and quantified on a Pharos FX densitometer using the Quantity One software (BioRad).

### Drug sensitivity screening of yeast cells

The screen was performed using 10 μM FTase inhibitor I on the barcoded yeast deletion strain collection generated by the *S. cerevisiae* deletion consortium [58] for all genes whose deletion has a viable phenotype in yeast. The screening was performed according to the procedures and protocols described in [55]. The cut-off for the hits was set at an average log_2_ ratio of 0.5 (p ≤ 0.05). Gene clustering and classification was performed using the GO Term tool of the SGD database (http://www.yeastgenome.org). Binning by biological process was performed with a maximal confidence setting as previously described [10]. Data mining was performed using NCBI databases (http://www.ncbi.nlm.nih.gov/pubmed/). Gene network analysis and network graphic representation was performed using STRING (version 8.3) software that collects data from known and predicted protein interaction databases freely available at http://string.embl.de. The interactions include direct (physical) and indirect (functional) associations; they are derived from Genomic Context, High-throughput Experiments, Coexpression Previous Knowledge. Confidence setting for data analysis was set at 0.7 (high confidence value).

### Human cell culture and drug treatments

Media, serum and reagents for tissue culture were purchased from GIBCO™ (Invitrogen). HeLa cells (ECACC) were grown in MEM supplemented with 10% foetal calf serum (FCS), 2 mM L-glutamine, penicillin, streptomycin and non-essential amino acids, at 37°C in 5% CO_2_. A375MM cells (kindly provided by Dr. Roberto Buccione, CMNS, Italy [28,29]) were grown in DMEM/F12 (1:1) supplemented with 10% FCS, 2 mM L-glutamine, penicillin and streptomycin at 37°C in 5% CO_2_. HT29 cells (ATCC) and A549 cells (ATCC) were grown in DMEM supplemented with 10% FCS, 2 mM L-glutamine, penicillin and streptomycin, at 37°C in 5% CO_2_. MCF7 cells (ATCC) were grown in MEM supplemented with 10% FCS, non-essential amino acids, insulin 10 μg/ml (Sigma), NaHCO_3_ 1 mM, penicillin and streptomycin at 37°C in 5% CO_2_.

### FTI compounds and treatment

The FTI-277 treatment of HeLa and A375MM cell lines for image analysis and proliferation assays were performed as previously described [10] with the indicated drug concentrations or, as mock reactions in parallel experiments, with the vehicle DMSO.

### PAK phosphorylation inhibitor

IPA3 was added, at the indicated concentration, alone or combined with FTI-277 or the vehicle (DMSO). Cells were incubated for the indicated times, as previously described [10].

HeLa cells for cell extract preparation were plated in a 6-well plate, left to attach overnight and treated with the indicated concentration of FTI-277 or vehicle (DMSO). After 48 h, cells were scraped off, collected, washed in phosphate-buffered-saline (PBS) 1× and lysed in a modified RIPA buffer [25 mM Tris–HCl pH 7.5, 150 mM NaCl, 1% NP-40, 1% Na-deoxycholate, 0.1% SDS, 1 mM EDTA, 30 mM β-glycerophosphate, 10 mM NaF, 5 mM Na orthovanadate, 1 mM PMSF, 1× Protein tablet inhibitor (Roche)]. Lysates were centrifuged for 10 min, 8000 × g, and then boiled in SDS-loading buffer prior to SDS-PAGE and immunoblot analysis, as previously described [10].

### Immunofluorescence

Immunofluorescence image analysis was performed in cells plated in 96-well Greiner-Bio-One plates using the Scan^R^ microscopy platform (Olympus) with a 20× objective as previously described [10,59]. Briefly, treated and control samples were plated in 96-well plates and left to attach for 24 h before drug(s) treatments. Drugs were added to the medium at the indicated concentration and incubation continued for the indicated times (4 h or 48 h). After treatment, cells were washed in PBS 1× and fixed in PBS 1× containing 4% paraformaldehyde for 10 min. Cells were permeabilized for 30 min in blocking buffer [0.05% saponin, 0.5% bovine serum albumin (BSA), 50 mM NH_4_Cl and 0.02% NaN_3_]. Fixed cells were then incubated with the primary antibody, washed three times in PBS 1× and incubated with the appropriate fluorescently-conjugated secondary antibody. The nuclei were stained with Hoechst, prior to being washed three times in PBS 1× and inspected. High content image analysis was typically based on data obtained from at least 3 wells/sample. Image segmentation and analysis was performed using the inbuilt Scan^R^ analysis software (Olympus) and based on a mask identifying the nuclei. The signal intensity values measured for each channel per sample were based on at least 12 images/well. Samples were swapped in the plate order in different biological replicates (typically n ≥ 2) to avoid local intensity signal drift as previously described [10]. All results are expressed as mean ± standard deviation (SD).

### Statistical analysis

Unpaired T-tests were used to assess differences between treatment vs. control samples. In the graphs the controls were normalized to 100 while changes in expression levels of treatments were analyzed as differences from normalized controls. P-values less than 0.05 were considered significant. Statistical analyses were performed using SAS® Language (Release 9.2. Cary, NC, USA; 2002–2008).

### Proliferation assays of human cells

MTS-based proliferation assays was performed using CellTiter 96® AQueous One Solution Cell (Promega) according to the manufacturer’s protocol. Typically, each cell line was plated in 5 wells and left to attach overnight in a 96-well plate (Falcon). Subsequently, they were treated with 5 μM FTI-277 and/or IPA3 at a concentration of 2 μM, 5 μM, or 7 μM. The IPA3 compound was added at the same time as the FTI-277 or the vehicle (DMSO) in parallel experiments. The number of living cells was measured at T = 0 and at T = 48 h. Briefly, 20 μl of Cell Titer was dispensed in each well containing 100 μl medium. The plate was incubated at 37°C in 5% CO_2_ sterile chamber for three hours, and the amount of formazan was measured reading the absorbance at 490 nm with a plate reader (BioTek Instruments). The results are the mean of three independent experiments.

## Abbreviations

FTI: Farnesyl-transferase inhibitor; FTase: Farnesyl-transferase; PAK: P21-activated kinase; PhoPAK: Phosphorylated p21-activated kinase.

## Competing interests

The authors declare to have no conflict of interests.

## Authors’ contributions

GP and ARW devised and performed the experiments and wrote the manuscript. MGM performed biochemical experiments. DDG technical assistance. GL statistical analysis. ABP and CB performed deletion library screening and related data analysis. All authors read and approve the final manuscript.

## Authors’ information

GP, post-doc. MGM, Ph.D. student. DDG, technician. GL, post-doc. ABP associate researcher. CB, Professor. ARW Aggregate Professor.

## Supplementary Material

Additional file 1: Table S1 The results of chemical profiling of yeast cells treated with FTase Inhibitor I. The table shows the genes whose deletion generates a hypersensitive phenotype to 10 μM FTase Inhibitor I treatment after chemical profiling of approximately 4700 barcoded yeast deletion strains. Genes discussed in more detail in this paper are highlighted in orange. Click here for file

Additional file 2: Table S2 Genes involved in transport and the genes that respond to chemical stimulus mediate the sensitivity of yeast cells to FTI. The table shows the biological processes associated with the 64 genes whose deletion generates a hypersensitive phenotype to FTase inhibitor I. The genes were binned using GO Slim Mapper binning by Biological Process (SGD, Saccharomyces Genome Database at yeastgenome.org). Each gene is listed by a gene identifier, the biological process assigned to the gene by SGD, the relative frequency of genes that have the same process compared to the total number of genes considered, and compared to the total number of genes that carry out that process in the whole yeast genome, and the names of the genes belonging to each group. Click here for file

Additional file 3: Figure S1 Cla4-GFP localizes like the wt Cla4 protein in BY4741 cells. Representative images of exponentially growing BY4741 wt cells carrying the plasmid Cla4-GFP pUG34 treated for 1 h with 10 μM FTase Inhibitor I (panel FTI) or with vehicle (panel Vehicle) as indicated in the text in the appropriate selective media. Microscopy inspection and image acquisition was performed as previously described using a 60× objective [10]. Click here for file

Additional file 4: Figure S2 A375MM cells are highly sensitive to 20 μM IPA3. A375 MM cells were treated for 48 h with the indicated compounds as indicated in Figure 5 and in Methods. % is relative to the vehicle arbitrarily considered as 100%. Error bars are means ± SD of 2 independent experiments calculated from 4 wells/sample. Click here for file

Additional file 5: Figure S3 Combined treatment of FTI-277 and IPA3 does not induce apoptosis in HeLa and A375MM cells. HeLa and A375MM cells were treated for 48 h as indicated in Figures 2, 3 and in Methods, and stained with Hoechst. Olympus Scan^R^ analysis software was used to calculate the number of apoptotic cells based on the total intensity Hoechst signal present within the nuclear region as described in [10]. More than 573 HeLa cells and 73 A375MM cells were counted per sample in each experiment. The graph represents the relative amount (%) of apoptotic cells in treated versus vehicle-treated cells, arbitrarily set at 100%. The graph shows the mean ± SD of 2 independent experiments, each run in triplicate (three wells per condition). Results of t-test are shown above the graph: ns: no significant deviation from vehicle, p-value >0.05; * p-value <0.05; ** p-value <0.01. Click here for file
