# Supplementary figures and images for: Combined p21-activated kinase and farnesyltransferase inhibitor treatment exhibits enhanced anti-proliferative activity on melanoma, colon and lung cancer cell lines
Source: Mol Cancer. 2013 Aug 6;12:88. doi: 10.1186/1476-4598-12-88 (PMC3765434; doi:10.1186/1476-4598-12-88)

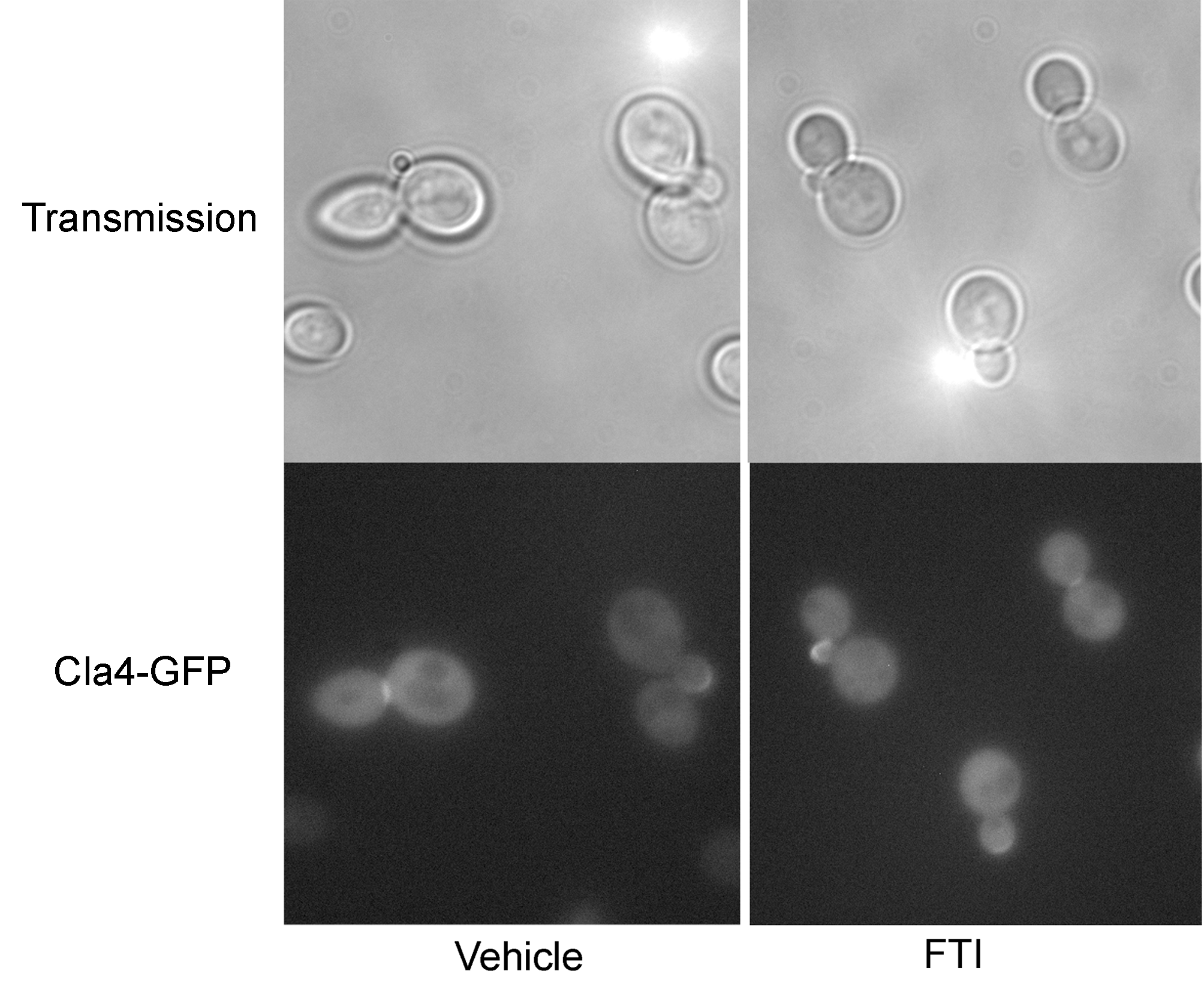

Supplement: Additional file 3: Figure S1 — Cla4-GFP localizes like the wt Cla4 protein in BY4741 cells. Representative images of exponentially growing BY4741 wt cells carrying the plasmid Cla4-GFP pUG34 treated for 1 h with 10 μM FTase Inhibitor I (panel FTI) or with vehicle (panel Vehicle) as indicated in the text in the appropriate selective media. Microscopy inspection and image acquisition was performed as previously described using a 60× objective [10]. [file 1476-4598-12-88-S3.tiff]

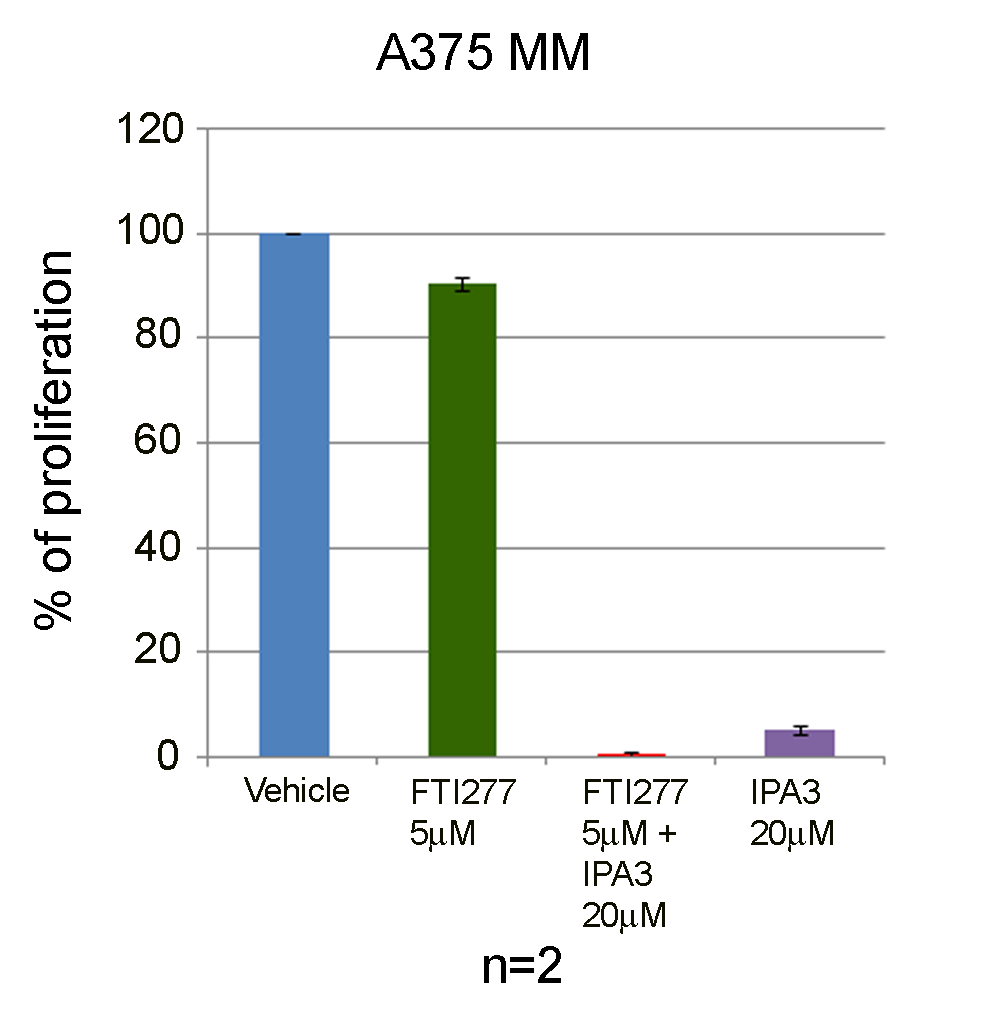

Supplement: Additional file 4: Figure S2 — A375MM cells are highly sensitive to 20 μM IPA3. A375 MM cells were treated for 48 h with the indicated compounds as indicated in Figure 5 and in Methods. % is relative to the vehicle arbitrarily considered as 100%. Error bars are means ± SD of 2 independent experiments calculated from 4 wells/sample. [file 1476-4598-12-88-S4.tiff]

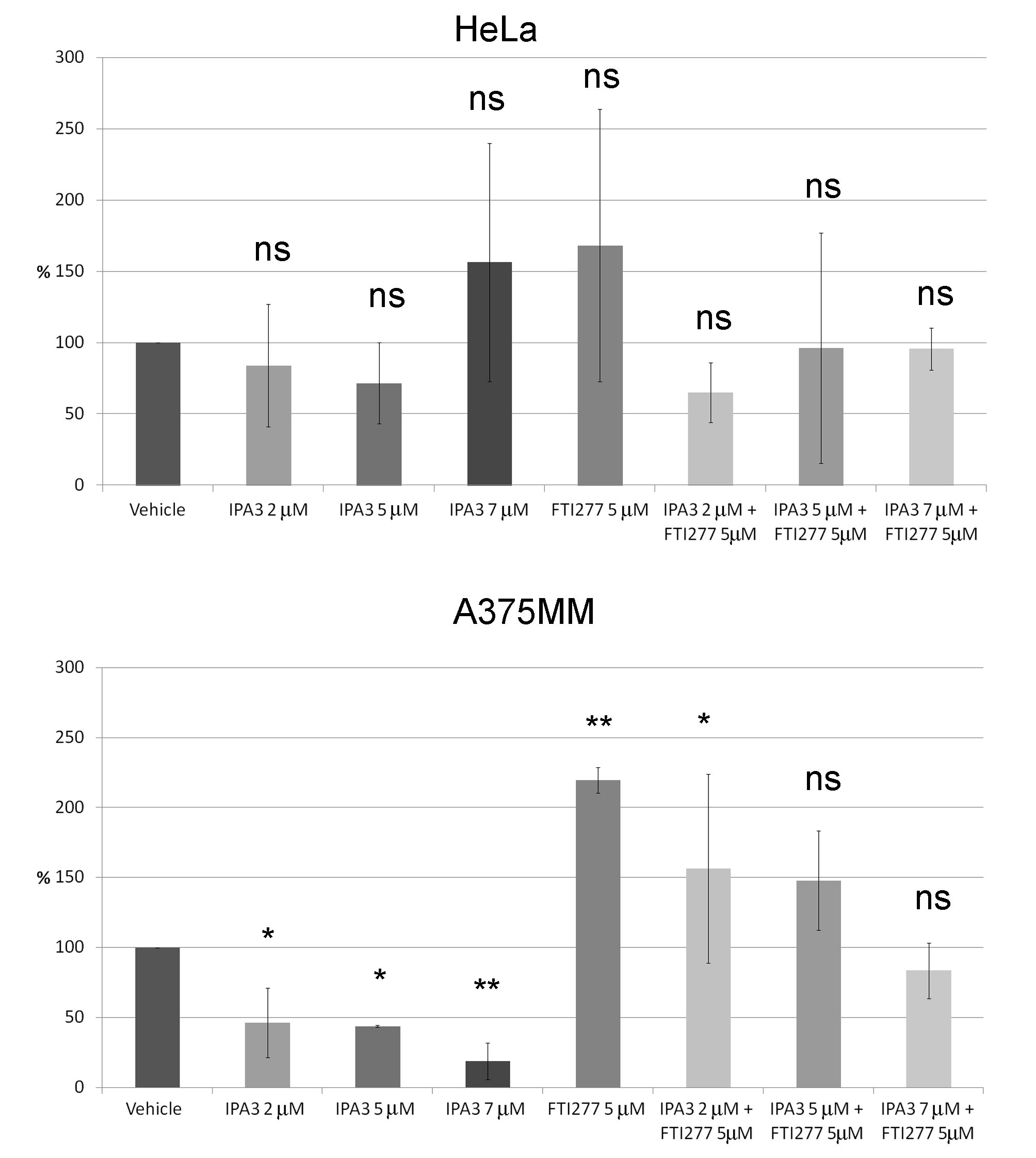

Supplement: Additional file 5: Figure S3 — Combined treatment of FTI-277 and IPA3 does not induce apoptosis in HeLa and A375MM cells. HeLa and A375MM cells were treated for 48 h as indicated in Figures 2, 3 and in Methods, and stained with Hoechst. Olympus ScanR analysis software was used to calculate the number of apoptotic cells based on the total intensity Hoechst signal present within the nuclear region as described in [10]. More than 573 HeLa cells and 73 A375MM cells were counted per sample in each experiment. The graph represents the relative amount (%) of apoptotic cells in treated versus vehicle-treated cells, arbitrarily set at 100%. The graph shows the mean ± SD of 2 independent experiments, each run in triplicate (three wells per condition). Results of t-test are shown above the graph: ns: no significant deviation from vehicle, p-value >0.05; * p-value <0.05; ** p-value <0.01. [file 1476-4598-12-88-S5.tiff]
